# A Treatment Model for Young Adults with Severe Mental Disorders in a Community Mental Health Center: The Crisalide Project and the Potential Space

**DOI:** 10.3390/ijerph192215252

**Published:** 2022-11-18

**Authors:** Maria Grasso, Rosalia Giammetta, Giuseppina Gabriele, Marianna Mazza, Emanuele Caroppo

**Affiliations:** 1 Department of Mental Health, Local Health Authority Roma 2, 00159 Rome, Italy; 2 Institute of Psychiatry and Psychology, Department of Geriatrics, Neuroscience and Orthopedics, Fondazione Policlinico Universitario A. Gemelli IRCCS, Università Cattolica del Sacro Cuore, 00168 Rome, Italy

**Keywords:** public mental health, recovery in mental health, cultural welfare and mental health, rehabilitation in mental health, early psychosis, personalized medicine in mental health, young adults

## Abstract

In line with priorities set by the Italian Ministry of Health and international literature, the “Crisalide project” provides specific care pathways aimed at young adults (YA) with severe mental disorders (SMD). As described in Materials and Methods, it consists of three lines of activity: transition to adult mental health services (TSMREE/CSM 17–19); Diagnostic, Therapeutic, and Assistance Pathways for Young Adults (PDTA-YA); high-intensity treatment center for young adults “Argolab2 Potential Space”. The aim of the study is to assess the results relating to the first three years of implementation of this clinical-organizational model (2018/2020) according to the process indicators identified by the ministry. Among the population aged 18–30 under treatment, results show increased prevalence (30%) and incidence (26%); 0% treatment conclusions due to the expiration of the conventional time limit; 0% involuntary hospitalizations (TSO); 0% STPIT hospitalizations; 0% repeated hospitalizations; 0% hospitalizations in the common mental disorders diagnostic group. Among the population of Argolab2 Potential Space, 45.4% have resumed studies; 40.9% have had a first work experience; 22.7% have obtained educational or training qualifications, and 18.2% live in independent houses. At a time when the academic literature underlines the terrible impact of the COVID-19 pandemic on this population, the present study confirms that specific treatment processes for young populations are a protective factor.

## 1. Introduction

The present study describes the organizational model and its theoretical framework, based on good practices in treating young adults (YA) with severe mental disorders (SMD) in a Community Mental Health Center (CSM) located on the outskirts of Rome. The aim of this study is to investigate the first results in model implementation, taking into account the three-year period between 2018 and 2020. Where possible, the results are compared with the indicators set up by the Italian Ministry of Health and data from the national Information System for Mental Health (SISM), year 2020. The main critical issues emerging from SISM concern poor planning and insufficiently differentiated requests.

The undifferentiated response is a key element in the historical path of psychiatry. In fact, the first psychiatric revolution that took place during the Renaissance overcame the magical religious beliefs about the origin of mental illness, leading it to a clinical–natural level. The second revolution, with the closure of the asylums and the complex cultural path underlying the reform (Law 180/1978, Law 833/1978), made possible the current community-based model of mental health care. The most recent literature data suggest how the timely interception of mental distress and appropriate interventions [1] represent the third revolution, as elements that often allow the resumption of the developmental pathway—disrupted by the first psychotic episode—and that avoid chronicization.

The Italian Ministry of Health defines “patients at onset”: “those under 30 years who had their first contact with the Mental Health Department (DSM) in the year of assessment and whose first ever psychiatric contact, if detected, is not older than two years” [2] (p. 6).

The average age of onset of mental disorders is between 15 and 35 years; however, the most important and recent cohort studies have found that the median age of onset is 22 or 23 years [3,4], that falling within the competence area of the CSMs. “The correlation between long DUP (duration of untreated psychosis) and poor outcome in the first years of illness has been systematically shown” [5] (p. 88), [6].

In the National Action Plan for Mental Health (PANSM), interventions on the onset of mental disorders are considered a priority need and, in its introductory part, a model is recommended that guarantees accessibility to services, early intervention and continuity of care, individualized projects, care pathways with different level of assistance according to the patient’s needs, and flexible services oriented towards needs and people. The recommended methodology emphasizes the importance of working with intervention projects that are differentiated and specific for each age group [7] (p. 4).

Indeed, for over a decade, the academic literature has underlined the need to improve the quality of care for young people who transfer from Children and Adolescent Mental Health Services (CAMHS) to Adult Mental Health Services (AMHS). It is a crucial element given the high risk of falling through the gap between services, with treatment interruption and severe consequences on outcomes [8,9]. On the other hand, “transfer to AMHS would arguably provide the benefit of appropriate mental health care and minimize harm from untreated disorder” [10] (p. 2).

In line with the above and with the PANSM indications, there are a number of key priority themes to consider in defining the treatment: timeliness and planning of the intervention, consequent implementation of the therapeutic pathway in its various articulations (pharmacological, psychotherapeutic, rehabilitative) and their necessary integration.

In terms of the intervention timing, it should be considered that the time period between 3 and 5 years following the psychotic onset is defined as “the critical period”, as it is deemed crucial for the future of those suffering from psychosis. The data show that what happens in this phase determines the course and prognosis of the disorder. All guidelines agree on continuous and intensive treatments in this phase [11].

Research on recovery in psychiatry has intensified in recent years. Certainly, the positive outcomes of intensive and specific interventions in first-episode psychosis have greatly contributed to increasing research in this direction. However, neither the organization of public services nor the treatments provided are in line with the latest findings. Several years ago, Saraceno already pointed out how, despite the diversity of diagnoses and the variety of theoretical models, treatments carried out by mental health services are few and always repetitive, and are not tailored to the real needs of patients. Therefore, we must reflect on this lack of specificity in psychiatry to understand how to intercept the real variables that change the real life of real people [12].

However, the above indications set up by the ministry find one of the main limits to their implementation in the serious lack of human resources that afflicts the current organization of Italian public mental health services. For 728,338 psychiatric patients assisted by specialist services, the total workforce within the public psychiatric units currently amounts to 28,807 units. Of these, 18.4% are doctors (psychiatrists or doctors with other medical specialties), 6.7% are psychologists; nurses are the most represented professional figure (44.8%), followed by auxiliary staff (11.2%), educators and psychiatric rehabilitation technicians (7.5%), and social workers (4.0%) [13] (p. 44). The shortage of personnel is also characterized by an even more serious shortage of personnel with specific skills: the percentage of doctors, psychologists, and psychiatric rehabilitation technicians is dramatically lower than the percentage of nurses.

“The available data on the activities of Mental Health Departments and services for neuro-psychic disorders in childhood and adolescence seem to indicate a lack of planning in care pathways. Such a situation, attributable to a not sufficiently differentiated request, creates the danger of a use of resources that is not appropriate to the complexity of the users’ needs. In many cases, users with severe disorders receive care pathways similar to those received by users with common disorders and vice versa. To this analysis we must add the evidence for a growing criticality related with the human resources in the services” [7] (pp. 6–7).

The PANSM identifies three clinical-organizational models capable of guiding the care process through interventions with increasing intensity depending on the patient’s needs: “(a) collaboration/counselling: a way of working, organized between DSM and general practitioners or between Hospital Unit of Child and Adolescent Neurology and Psychiatry (NPIA), pediatricians, psychological or social services and schools, for users who do not require ongoing specialist care; (b) specialist intervention: a treatment pathway for users who require specialized treatment but not complex and multi-professional interventions; (c) taking charge: an integrated treatment pathway for users who have complex needs and require a multidimensional assessment and the intervention of different professional profiles” [7] (p. 7). These three models were applied to all CSM patients.

The “Crisalide” project was launched in 2018 to reduce the gap between the ideal treatment model proposed by the guidelines and the actual organizational reality of public mental health services, taking into account the high criticalities of the social context in which it takes place, as indicated by WHO [14] and addressed later. Its main objective is to reduce early institutionalization and the consequent chronicization of mental disorders. In order to identify treatment pathways that are appropriate to SMD and respect the priorities of treatment for the YA age range, the Crisalide project is structured along three lines of activity:

- Transition to adult mental health services (TSMREE/CSM 17–19);
- Diagnostic, Therapeutic, and Assistance Pathways for Young Adults (PDTA-YA);
- High-intensity treatment center for young adults “Argolab2 Potential Space”.

## 2. Materials and Methods

### 2.1. Transition to Adult Mental Health Services (TSMREE/CSM 17–19)

In our DSM organization, services for children and adolescents are aimed at the 0–18 age range. Therefore, the transition to adult services coincides with a stressful phase that involves the entrance in the adult world and the corresponding consolidation of the self. “This can be a source of considerable stress for young people and their families, as family and social roles must be renegotiated to accommodate the young person’s developing sense of autonomy” [15] (p. 5).

A first critical issue arises from the different organization of CAMHS and AMHS. In fact, CAMHS mainly follow the criterion of the age range (0–18 years) and not that of the specificity of treatment needs. As a major study points out, “neurodevelopmental disorders were the most frequent diagnostic group (up to 81%) for people seen at CAMHS from 46% of countries”. Hence, “the heterogeneity in resource allocation does not seem to match the epidemiological burden for all diagnoses including physical and intellectual disabilities, neurological disorders, substance abuse” [9] (p. 715), while AMHS deliver differentiated services for these disorders. Therefore, to guarantee a treatment pathway that is the most appropriate for diagnosis and implemented in appropriate services, there is a need to reassess cases at the age of 18, so that a greater diagnostic accuracy is gained.

Another critical issue, specific to our CSM catchment area, is the rate of children and adolescents placed by the social services in residential care: it is the highest rate in Rome [16] and implies the occurrence of early institutionalization. Although it happens because of mainly socio-economic reasons, it represents a risk factor on account of which, due to a lack of adequate resources, the request for placement into residential care is repeated even after the age of 18 and young people improperly come to the attention of psychiatric services. Furthermore, as indicated by the Ministry of Health: “particular attention must be paid to the transition of under-age subjects when they turn 18 years of age. In this regard, specific protocols must be developed between NPIA services and DSM services so that the transition occurs in the least traumatic way possible. Transition must be made at the time most suitable for the individual, even if it occurs after the age of 18. When the conclusion of the care relationship is expected to happen within 12 months, it is necessary to continue the treatment at the NPIA services even after the age of 18. In planning the transition, analyzing the opportunity of implementing a care plan outside the Adults Mental Health Services, using other resources, is also recommended” [2] (p. 29).

To pursue these objectives, a TSMREE/CSM protocol has been defined for the 17–19 age range. The protocol has been enriched with further key elements. Indeed, the CAMHS contact person and AMHS contact person have regular monthly meetings to discuss and monitor the pathway of patients who are about to turn 18 years of age. The need for collaboration/counselling, specialist intervention, or taking charge is assessed through an accurate presentation of the life story, the therapeutic pathway, and the psychometric assessments carried out. If the treatment criteria specified by the Ministry of Health for treatment at the CSM (addressed later) are met, an appointment is made with the patient and the CSM specialist who will be the clinician of that patient. If the adolescent does not meet the criteria for treatment at the CSM (non-psychiatric diagnoses), the CAMHS contact person will refer the patient to the most appropriate adult service. In case of doubt, a further diagnostic examination is carried out; psychodiagnostic or instrumental investigations are requested and, if needed, a joint assessment interview involving the patient, the CAMHS contact person and the AMHS contact person is implemented. If the adolescent is reluctant or has communicated to the clinician practical difficulties in accessing the adult service, one to three joint interviews are planned to manage any questions or obstacles and offer support for change, following the specific recommendations of NICE [17].

Cases between the ages of 17 and 18 that come to the attention of the TSMREE for the first time or are hospitalized in the NPIA wards and whose treatment is unlikely to end within 12 months are referred to the CSM. The joint assessment is carried out and the taking over by the CSM occurs, if appropriate, at the time of the agreed discharge. This process makes it possible to avoid a further transition due to the bureaucratic limit of 18 years. In fact, the TSMREE should intervene with the adolescent as a minor, but it would refer the patient to the CSM after a few months, given the age of majority and the criterion of continuity of care.

### 2.2. Diagnostic, Therapeutic, and Assistance Pathways for Young Adults (PDTA-YA)

Within the three care models applied to all CSM patients, the Diagnostic, Therapeutic, and Assistance Pathways for Young Adults (PDTA-YA) attempt to integrate the need for treatment with the priority for intensive and specific interventions for SMD in young adulthood.

The inclusion criteria are as follows:

1. Aged between 18 and 30 years;
2. Absence of moderate or severe mental retardation;
3. Exclusion of organic psychoses;
4. Absence of substance addiction;
5. Fulfillment of the criteria for one of the following ICD-9 diagnoses: (1) schizophrenia spectrum disorders; (2) affective psychosis, and (3) severe personality disorders.

Regarding severe personality disorders, particular attention is paid to the indications provided by the Ministry of Health guidelines: “abnormal behaviors are only one aspect of severe personality disorders, that, in the absence of specific psychopathological alterations, do not present diagnostic value nor are they indicative of usefulness of psychiatric interventions” [2] (p. 27).

The PANSM guidelines recommend to include severe personality disorders with repeated self-harm and/or suicide attempts; at least three hospitalizations and/or placements in residential facilities within a year, or one hospitalization lasting more than one month; previous care in NPIA and repeated school and/or work failures; and impaired social and/or interpersonal functioning. Regarding borderline personality disorder, “studies on early interventions suggest that the timely initiation of treatment may reduce the most serious manifestations and improve outcomes” [2] (p. 32). These PANSM indications concerning treatment pathways allow to overcome the requests for social control addressed to psychiatry and to carry out appropriate and specific interventions based on the most current knowledge. Furthermore, “there is no single feature that differentiates SIP (substance-induced psychosis) from PPD (primary psychotic disorder) in a young patient using substances, and it is often only the presence or absence of psychotic symptoms during prolonged periods of abstinence–if this can be achieved—that will definitively determine the need for ongoing treatment of psychosis. That said, there are some clinical features that may point to one etiology over another” [18] (p. 7). As a result, the differential diagnosis between SIP and PPD is required to implement the right pathways and avoid chronicization due to an ongoing psychiatric treatment of psychotic symptoms induced by substances.

Patients who fulfill the criteria have an initial interview with the psychiatrist within 1–7 days, depending on the urgency.

Clinical cases are discussed in fortnightly 90 min meetings, structured as an accredited Continuing Medical Education course (ECM). All the professionals of the service participate in these meetings. The clinician proposes a case to talk about based on two criteria: 1. development of PDTA-YA after the first interviews, or 2. reassessment of PDTA-YA, for cases already in treatment and in need of new therapeutic interventions. The case presentation is required to focus on the individual and family history, seen in relation to the psychic development pathway.

In fact, the name of the project (“Crisalide”, chrysalis) refers to Winnicott’s question: “Is it possible to describe not only the dragonfly, but also the process of metamorphosis and, indeed, the chrysalis itself? That would indeed be good” [19] (p. 250). The choice of the name condenses several observations from which several methodological choices arise. The first methodological choice is the specificity of this age group. The pioneer of adolescent psychoanalysis, Peter Blos, had defined adolescence as a “second individuation”, referring to the developmental process (separation-individuation process) [20]. His insight is currently confirmed by neuroimaging showing that “adolescence is one of the most dynamic events of human growth and development, second only to infancy in terms of the rate of developmental changes that can occur within the brain. In fact, there are characteristic developmental changes that almost all adolescents experience during their transition from childhood to adulthood. It is well established that the brain undergoes a “rewiring” process that is not complete until approximately 25 years of age” [21] (p. 451), [22]. Therefore, the image of the chrysalis in Winnicott’s words represents the theoretical background underlying the ongoing attention to the psychic process in the development of the subject; this theoretical framework helps to take into account the specific dynamism of the adolescent/young adult and to understand the psychopathological dimensions. In this regard, the concept of subjectivation is to be considered a valid point of reference in the process approach to the understanding of complex psychic phenomena. Indeed, the term subjectivation represents an important encounter between philosophy and psychoanalysis; in fact, it connects Foucault’s definition of the normative devices of knowledge-power that act on subjective life by providing it with concrete ways of self-designation, already present in society and culture of a given era [23], and the definition of subjectivation suggested by adolescent psychoanalysis, that is a process of knowledge and self-definition of the self [24]. This theoretical link offers great possibilities for the development of a psychosocial perspective in the various articulations of the path from adolescence to young adulthood; in this developmental pathway, the ongoing process of integration of previous achievements and new skills, due to the growth potential and the innate maturation push, tends to guarantee the continuity of the Ego experience in order to facilitate the emergence of a stable sense of social self [25]. SMD are clear and serious expressions of the impasse of this troubled process. These theoretical assumptions, just mentioned here for reasons of synthesis, are needed to provide further knowledge tools that facilitate the understanding of psychic events and help to formulate a hypothesis about when the breakdown that blocked the developmental pathway occurred. Hypotheses based on knowledge and understanding of psychic phenomena are a valuable contribution to reducing the levels of anxiety that arise between a mental health professional and a patient with a severely altered relationship with reality. This approach enables the clinician to overcome the narrow view of symptoms or behaviors as something to intervene on using a mere psychopharmacological treatment or hospitalization, and opens a more complex perspective that may realize a biopsychosocial model as defined by the WHO. This is even more important if we consider the heterogeneous training of professionals working in mental health services, a heterogeneity due to roles and functions (psychiatrists, psychologists, nurses, and social workers) and to the theoretical orientation (cognitivism, phenomenology, family systems therapy, psychoanalysis, etc.). Creating a common culture oriented to the specificity of the intervention, within a heterogeneous and generalist cultural environment, is a central factor of cohesion of the working group that facilitates communication through notions expressed in a shared language understandable to all.

Furthermore, sharing within the group of professionals helps to reduce the experiences of loneliness and helplessness that are often reflected between patient and clinician; these experiences are even more negative when they concern young patients, especially in circumstances such as suicide attempt, self-harm, and destructive acting out. The maintenance of the working group, the culture of organizational well-being, and the specificity of training are issues that, although important, are still too neglected in public health institutions. As reported by a huge body of research, they are protective factors for care professionals in the prevention of burnout and also have a positive impact on patients, improving treatment outcomes [26,27,28,29].

PDTA-YA include two types of projects: combined and integrated, to be built according to individual needs. The combined project consists of an individual psychological path and psychopharmacological treatment, based on specific needs and aimed at achieving the best possible balance between efficacy and side effects. It is widely confirmed that the metabolic and extrapyramidal effects represent the main causes of pharmacological treatment interruption and of relapses that worsen the prognosis; “significant differences between treatments were found in the categories of sleepiness/sedation, increased sleep duration, akinesia, weight gain, ejaculatory dysfunction, extrapyramidal-symptoms, and amenorrhea” [30] (p. 218). Indeed, physical and intellectual performance and body image play a central role in this period of life in which the representation of the body self and the representation of the social self are consolidated. Attention to the most dynamic time in life after infancy, in terms of rate of developmental changes that can occur within the brain [21], must correspond to attention to information, discussion, and regular renegotiation of pharmacologic prescription. As indicated by the National Institute of Mental Health, “guideline-based use of medication optimizes the speed and extent of recovery, as well as acceptance of pharmacologic interventions. Medical care of young people during the early stages of mental illness is considerably different in style and content compared to approaches used in older individuals with established illness” [31,32].

Historically, “standard treatment (ST) for psychosis consists primarily of antipsychotics, hospitalization, social rehabilitation, and different types of supportive therapy” [33] (p. 1). We know that antipsychotic drugs have only moderate effects on positive symptoms and no demonstrable effect on negative symptoms [34]. Regarding the individual psychological path, we have decided to propose weekly interviews in order to guarantee continuity and regularity and facilitate the building of a good therapeutic alliance, according to the indication for intensive treatment and the continuity of the intervention. Concerning the theoretical framework, most of the CSM therapists have a psychoanalytic or phenomenological background, while a small part of the team is made up of cognitivist or family systems therapists. Beginning with a major Cochrane systematic review [35], the most recent studies comparing the efficacy of different psychotherapy models agree that there is no evidence to support one model having a greater impact than another on positive outcome [36,37,38,39,40,41,42]. On the other hand, all studies and treatment indications agree on the efficacy of specific and integrated treatment models for the young adult population with SMD. Therefore, the combined treatment project (psychopharmacological and psychological) represents the minimum treatment proposal on which, depending on the personalized needs, other pathways and professional figures are inserted in order to outline an integrated treatment project: family therapy; parental counselling; support for autonomy; social service and possible referral to the high-intensity treatment center for young adults described in the following paragraph.

### 2.3. High-Intensity Treatment Center for Young Adults “Argolab2 Potential Space”

The PANSM indicates that most patients at onset require “a care pathway that may however be characterized by different assistance intensities depending on the disorders, contexts, and developmental phases, and not only based on complexity and severity. As a consequence of the above, longitudinal monitoring of development is much more frequent than a single episode of care, because developmental disorders change in time and over time along complex and specific lines, and rehabilitation is an indispensable component of the care process” [7] (p. 8). The PANSM considers “rehabilitation” as strictly related to “contexts and developmental phases”.

These observations overlap with the analysis of the real-life context of the population we are dealing with. This area, located on the eastern outskirts of Rome, is the most disadvantaged in Rome and other Italian cities, as shown by several social indicators. It has 257,000 inhabitants, 35% of whom are under 30 years, an average age lower than that of the other Roman municipalities. Between 27 and 30% of the population has a primary school certificate or no educational qualification and only 5% are graduates; the cultural opportunities (cinemas, theaters, and libraries per percent of residents) are 0.01–0.07%; 6% drop-out of lower secondary school; the rate of young people aged 15 to 29 unemployed or out of the educational system (NEET + 15%), and the rate of families with economic and social hardship (up to 7.5%) is at a maximum [43].

It is well established that “the early stages of psychosis, including the prodrome, often feature educational/occupational difficulties and various symptoms and signs, that can render or keep youths ‘Not in Employment, Education or Training’ (NEET). Conversely, NEET status itself may increase risk of illness progression and impaired functioning, and impede access to appropriate services for psychosis” [44] (p. 1401). There are no data that accurately establish a causal relationship between low social indicators and psychosis, but there is strong evidence for an unfavorable correlation with the course of the disorders. Furthermore, data from the academic literature agree in identifying the interruption in studies, social withdrawal, and the loss of interpersonal and cognitive skills as dramatic outcomes of psychosis when not treated in an intensive and timely manner, and point out that the earlier the onset is, the worse the outcomes are.

In 2020, these remarks led to transforming an old day center into the “Argolab2 Potential Space for Young Adults”, a semi-residential facility, open eight hours a day and managed by a team made up partly of third sector employees, partly of public employees.

Argolab2 offers various activities such as support for educational and employment attainment, carried out through personalized consultations and individual lessons, in contact with public schools. “Supported Employment/Education (SEE) services help clients return to work or school and achieve their personal goals. Emphasis is on rapid placement in a work or school setting combined with coaching and support to ensure success” [45] (p. 120). To foster greater autonomy, Argolab2 supports study and employment recovery through consultancy implemented at the office, with no coaching in the actual setting in which these activities take place. When it is necessary to provide coaching in the actual work environment, internship projects are carried out with the assistance of the CSM social workers.

A band was born from music theory lessons and instrumental practice; journalism gave rise to a monothematic four-monthly magazine that reflects youths’ curiosity about current events and their context of life; a web radio created podcasts and carried out interviews. Other activities are theater, street art, video making, sport, and care for the environment. The teaching of the main disciplines is provided by professionals in the specific sector (expert artisans or artists) and not by mental health professionals; the latter mainly deal with difficulties with interpersonal relationships, facilitate autonomy, and monitor progress or any critical issues encountered by youths in their real life. Each activity is aimed at promoting a real learning of skills, according to personal inclinations and curiosities, and at encouraging and supporting new experiences and the exploration of cultural elements. As underscored by a WHO recent scoping review [46] (p. 4): “the aesthetic and emotional components of arts activities can provide opportunities for emotional expression, emotion regulation and stress reduction. Emotion regulation is intrinsic to how we manage our mental health. Cognitive stimulation when engaging in the arts can provide opportunities for learning and skills development, and it is not only associated with a lower risk of developing dementias but also interrelated with mental illness such as depression. Social interaction while participating in the arts can reduce loneliness and lack of social support, which are both linked with adverse physiological responses, cognitive decline, functional and motor decline, mental illness and premature mortality”.

This feature strongly shapes the activities of Argolab2 Potential Space for Young Adults, whose name stems from Winnicott’s concept of Potential Space: “where there is trust and reliability is a potential space, one that can become an infinite area of separation, which the baby, child, adolescent, adult may creatively fill with playing, which in time becomes the enjoyment of the cultural heritage. The special feature of this place where play and cultural experience have a position is that *it depends for its existence on living experiences*, not on inherited tendencies” [47] (p. 108). “Playing and cultural experience are things that we do value in a special way; these link the past, the present, and the future; *they take up time and space* […] The baby’s confidence in the mother’s reliability […] makes possible a separating-out of the not-me from the me” [47] (p. 109). The chosen name summarizes the attention to the developmental pathway that tends towards the continuity of internal experience, arriving at subjectivation and autonomy, seriously undermined in psychotic experiences.

YA participate in individual and group meetings. Group participation is crucial when it comes to organizing cultural events aimed at their community and its enrichment. The community sees YA active in proposing what they have learned and returns them a more cohesive and skilled image of themselves.

Argolab2, the other part of the name, is connected to the image of the institutional container in which this experience may occur. It takes the symbol of the ship *Argo* from mythology, not so much for the challenge that led the young anti-heroes to fetch the Golden Fleece, as for the specific features of the ship construction. In fact, the ship Argo was built following the detailed indications of the goddess Athena to be resistant, fast, and able to actively participate in the adventures of the large crew [48]. Thus, the chosen name intends to express the desire that skills may be made available to everyone, in coherence between the theory and practice of the organizational model, by a public institution capable of supporting, holding, and responding in a dynamic way.

Referrals to Argolab2 are decided during the clinical discussion meetings described in the previous paragraph and concern all patients of the 18–30 age group under treatment with a specific attention to personal development needs, according to the Crisalide project, and to situations of severe social withdrawal and disruption of everyday activities. Argolab2 is located in an apartment in public housing, among others houses, not far from the CSM.

In an initial interview, the service manager provides the young adult with all the information on the functioning of Argolab2 and on current projects, and proposes a week of exploration and participation to the various workshops and a second appointment to discuss which interests have emerged and decide the frequency of participation (from 2 to 5 times a week).

During the restrictions imposed by the COVID-19 pandemic [49], Argolab2 maintained regular activities in compliance with the anti-COVID-19 rules, providing YA with devices to guarantee remote activities and allow them to resume or continue their studies.

In order to prevent the institutionalization related to the stagnation of care pathways, the duration of treatment at Argolab2 is 5 years for each young adult, a time consistent with what has been defined *the critical period* following the psychotic onset.

Once a week, there is a meeting of the service team that involves coordinators and nurses and during which, in addition to organizational issues, information on the life stories of the YA recently referred to Argolab2 and the observations on the pathway of those already attending it are shared. Updates on the pathways are regularly shared with the CSM clinicians. Once a month there is an extended meeting that also involves the expert artisans/artists and focuses on the activities and events to be carried out. Expert artisans/artists do not know either the diagnoses or the stories of the YA they teach; thus, learning focuses on young adults’ resources and not on their disorder and takes place in a relationship that is as natural as possible; furthermore, such a methodological position guarantees YA respect for their privacy and allows them to be free to make themselves known according to what they wish to share. The training of the Argolab2 team occurs every two months and consists of organizational consultancy aimed at acquiring an understanding of the organizational and social group dynamics, the interaction between tradition, innovation and change, and the relationship between the organization and its social context. These meetings may help a team highly heterogeneous in terms of training and roles to focus on goals and enhance the creative potential that lies in the differences between group members, according to the Tavistock approach.

## 3. Results

The International Classification of Diseases, 9th edition (ICD-9-CM; World Health Organization), was used to assign psychiatric diagnoses. Diagnoses were grouped according to criteria corresponding more to the model implemented. The first three diagnostic groups consisted of the severe diagnoses covered by the model described (Table S1). The other groups received other treatment pathways and was included for comparison, in addition to some data concerning the total population of CSM patients.

### 3.1. Transition to Adult Mental Health Services (TSMREE/CSM 17–19)

The mental health information system used by the CSM does not allow to digitally collect information about the line of activity 17-19 years; therefore, data shown in Table 1 come from a paper database. In 2018, there were 21 referrals from the TSMREE, and, once they reached the age of majority, the CSM took charge of them all. In 2019, there were 13 referrals: the CSM took charge of 6 (46.2%) of them; 4 (30.7%) had a joint assessment, undertaken by TSMREE and CSM together, and conclusion of treatment by the age of 19, and 3 (23.1%) were referred to other services because they did not have a psychiatric diagnosis. In 2020, there were 12 referrals from the TSMREE: the CSM took charge of 8 (66.7%) of them; 1 had a joint TSMREE/CSM assessment and conclusion of treatment by the age of 18, and 3 (25%) were referred to other services because they had disorders other than psychiatric ones. The average age of youths referred by TSMREE decreased slightly from 2018 to 2020: from 19 to 18.4 years.

### 3.2. Diagnostic, Therapeutic, and Assistance Pathways for Young Adults (PDTA-YA)

Table S2 shows data on each year treated prevalence, distributed by gender and diagnosis; medicolegal medicine consultations are excluded. “Treated prevalence in a certain year is given by the number of patients with at least one contact during the year with the services of the Mental Health Departments” (SISM) [13] (p. 50).

The number of prevalent patients aged between 18 and 30 and with severe diagnosis increased by 30% (from 49.5% in 2018 to 79.3% in 2020) and the categories of no psychiatric disorder and unknown diagnosis disappeared. In 2018, the categories of common mental disorders, other disorders, no psychiatric disorder, and unknown diagnosis accounted for 50.4% of the treated patients in this age group. The gender distribution of the different diagnostic groups in the YA population reflected the epidemiology of the population data: prevalence rates were substantially the same in males and females, with severe diagnoses more frequent in males, except for affective psychosis that had higher rates in females; the female gender was less represented here due to the earlier onset in males. The mean age was 25.

Distribution by nationality of treated prevalent YA patients remained constant in the given period. In 2018–2020, Non-Italian YA patients were on average 18.7%. (Table S3)

Treated incidence concerns patients who had contact with the CSM for the first time ever in the year (new cases) [13] (p. 66). Data refer to incident population that actually received interventions, excluding medicolegal medicine consultations (Table S4). Incident YA patients with SMD who were treated at the CSM showed an average increase of 26%, in 2019-2020. In 2018, 62.2% of incident YA patients had diagnoses not belonging to SMD groups.

In the given period, the percentage of incident YA patients out of the total population of incident patients increased by 9.3%: in 2020, 28.8% of incident patients were under the age of 30. The percentage of incident YA patients with SMD increased by 6.1% over the three years. The mean age of the total incident population decreased by 10.5 years, while the mean age of the incident YA patients remained constant, which was 23 years (Table S5).

**Figure 1:**
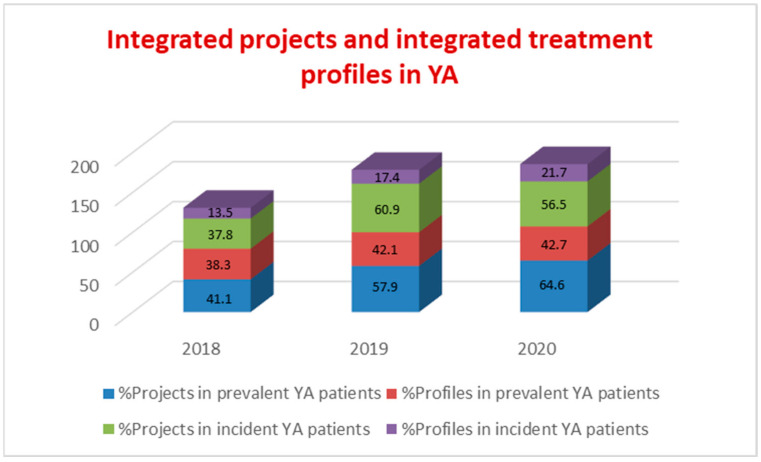
Prevalent YA patients and incident YA patients—Integrated projects and integrated treatment profiles.

In the comparison between the three years, both the therapeutic projects (planning of a care pathway based on the patient’s needs and agreed with the latter) and the treatment profiles (actual care pathway measured through the interventions carried out) of an integrated type aimed at YA with SMD increased, both in the incident population and prevalent population, as shown in Figure 1.

**Figure 2:**
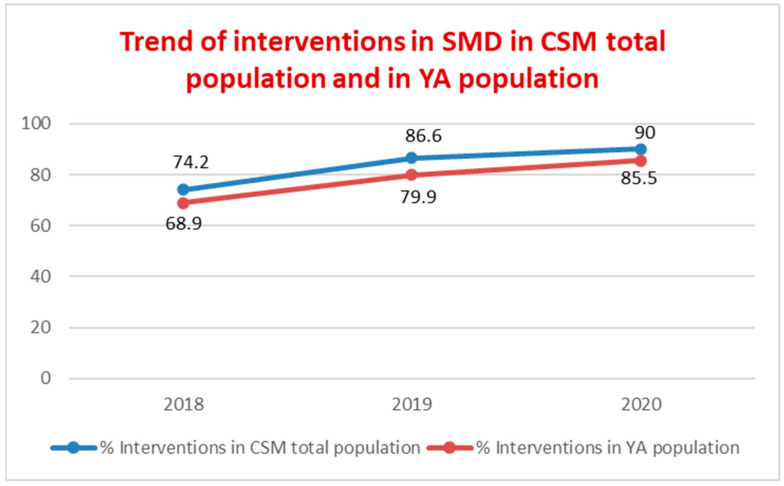
Trend in percentage of psychiatric, psychological, psychoeducation, or social interventions delivered to CSM total population with SMD and to YA population with SMD per year.

Over the three years, the percentage of interventions delivered to total population with SMD and YA population with SMD increased, as shown in Figure 2.

**Figure 3:**
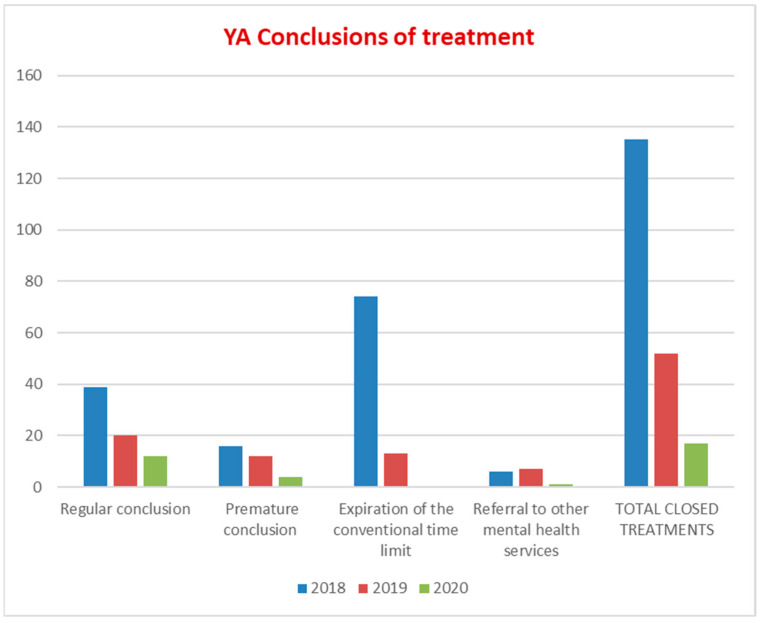
YA conclusions of treatment.

In our mental health information system, conclusions of treatments due to the expiration of the conventional time limit refer to conclusions occurring because for a 90-day period no intervention has been delivered to the patient and recorded in the information system. Overall, conclusions of treatments in prevalent YA patients fell by 24% (from 51% in 2018 to 27% in 2020); conclusions due to the expiration of the conventional time limit were reduced by 30% (from 51% in 2018 to 21% in 2020). No patient with SMD had a conclusion of treatment due to the expiration of the conventional time limit or referral to other mental health services due to conditions not falling under the competence of the CSM (Figure 3).

In the Italian mental health system, the SPDC (General Psychiatric Unit for Diagnosis and Care) is a psychiatric ward within the general hospital where voluntary and involuntary inpatient treatments are implemented 24 h a day. The STPIT (Facility for Territorial Intensive Psychiatric Treatment) delivers inpatient treatments to those patients who, at the time of discharge from SPDC, are deemed to have to continue a voluntary hospitalization, in a still very complex healthcare context; STPIT is also intended for the voluntary hospitalization of people whose conditions are less severe than those admitted to SPDC, but who in any case require hospital care.

**Figure 4:**
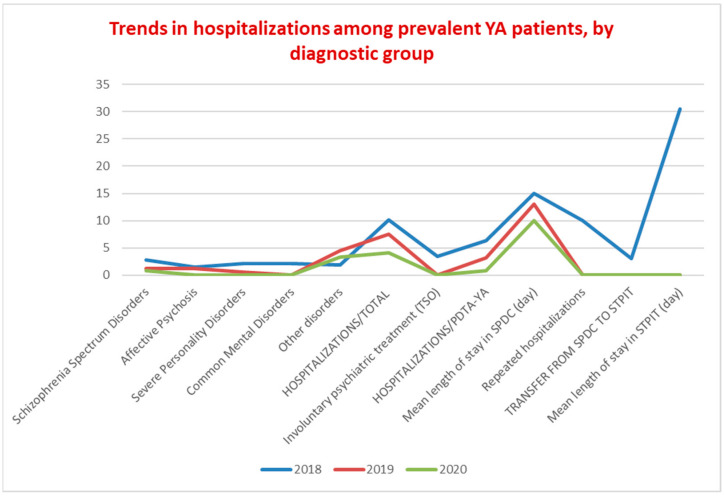
Trends in hospitalizations among prevalent YA patients, by diagnostic group.

Figure 4 shows the trends in hospitalizations among the YA population in the given period. In 2018, the total number of hospitalizations in SPDC was 29, of which 1 was a TSO (involuntary psychiatric treatment). In Italy, TSO is governed in accordance with articles 33, 34, and 35 of Law 833/1978, according to which a citizen may be forced to health interventions in hospital against their will “only if there are psychiatric alterations such as to require urgent therapeutic interventions, if the latter are not accepted by the patient, and if there are no conditions and circumstances that allow to adopt timely and suitable out-of-hospital health measures”. The distribution by diagnosis of the number of hospitalizations was homogeneous. It is worth noting that, from 2018 to 2020, the trend in YA hospitalizations had a progressive reduction of up to 6%. In 2018, 10 patients with SMD received multiple hospitalizations in SPDC and 3 were admitted also in STPIT (mean length of stay in STPIT 30.5 days); in 2019 and 2020, there were no repeated hospitalizations, no hospitalizations in STPIT, nor TSOs. The mean length of stay in SPDC progressively decreased from 15 to 10 days. Hospitalizations of patients belonging to the common mental disorders diagnostic group were 6 in 2018 and fell to 0 in the following two years.

### 3.3. High-Intensity Treatment Center for Young Adults “Argolab2 Potential Space”

Data from the high-intensity treatment center for young adults *“Argolab2 Potential Space”* refer to the period from 2020 (year of its launch) to July 2022. Resumption of school and/or work and housing autonomy are data not digitally collected by our current mental health information system and were recorded in a paper database. In the given period, the total number of patients accepted at Argolab2 was 22. The mean age was 24 years. Males were 41% and females were 59%; diagnosis distribution by gender corresponded to epidemiology. Italian patients were 63.6% and non-Italian patients were 36.4%. As for the diagnostic groups, 50% belonged to the schizophrenic spectrum, 27.3% to severe personality disorders, and 22.7% to affective psychosis (Table S6). The Mean Global Assessment of Functioning scale (GAF) score at baseline was 55.

**Figure 5:**
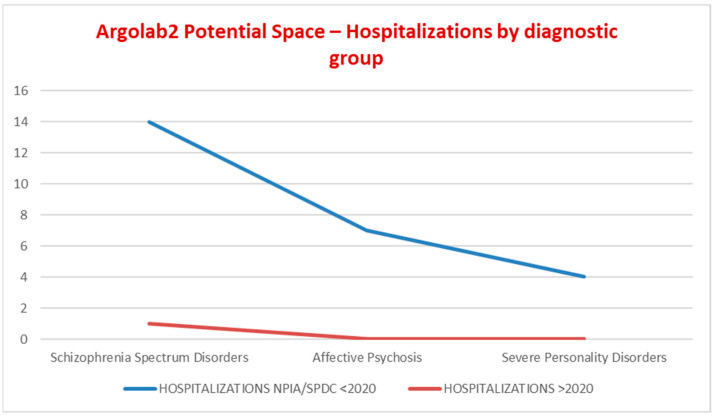
*“Argolab2 Potential Space”*—Hospitalizations by diagnostic group.

Figure 5 shows the trend in hospitalizations. Before accessing Argolab2, the total number of hospitalizations in NPIA and/or SPDC received by the 22 young patients was 25: 3 had never been admitted to a psychiatric ward, 19 had been admitted one to five times to NPIA and/or SPDC (114%). After accessing Argolab2, one patient was admitted in SPDC once (4.5%).

**Figure 6:**
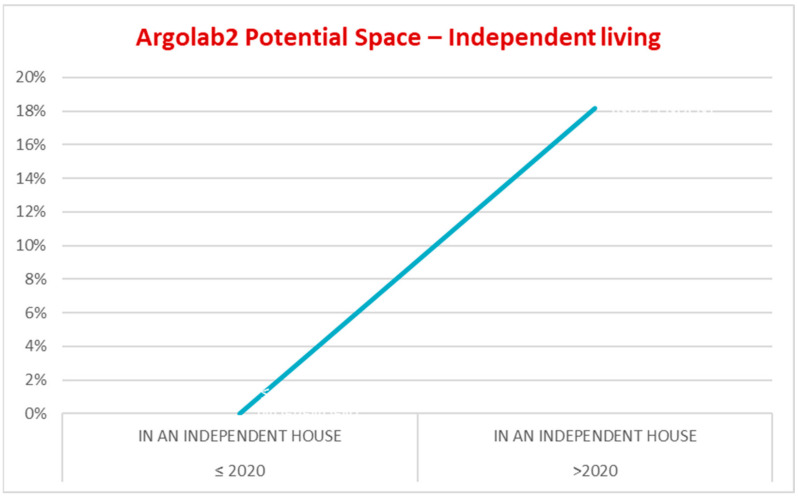
*“Argolab2 Potential Space”*—Independent living.

**Figure 7:**
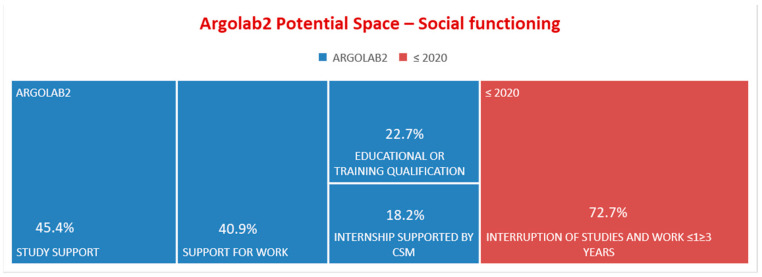
*“Argolab2 Potential Space”*—Social functioning.

Before attending Argolab2, 100% lived with their family of origin; after 2020, 18.2% lived independently (Figure 6). At the time of accessing Argolab2, 72.7% had interrupted their educational courses and work activities for a period between 6 months and 3 years. After 2020, 45.4% resumed their studies; 40.9% had their first work experience; 22.7% obtained educational or training qualifications; 18.2% started an internship supported by CSM (Figure 7).

## 4. Discussion

The present study explored the trends in the main treatment indicators to assess the efficacy of the clinical-organizational model implemented as described in the Materials and Methods paragraph.

Data on TSMREE/CSM 17–19 showed that, in 2018, 100% of the referred cases made a transition from TSMREE to CSM at age 18, regardless of diagnosis. The implementation of the model showed that, in 2019, 30.7% had a joint TSMREE–CSM assessment and consequent conclusion of treatment by the age of 19. In 2020, 25% were referred to other services after an in-depth diagnostic assessment. That demonstrates a more specific and differentiated percentage trend of referrals, as suggested by the guidelines. In addition, as required by an early intervention [1], a reduction from 19 to 18.4 years in the mean age of cases in the transition phase was observed. In 2019 and 2020, due to a strong collaboration between the two services, as advocated by the guidelines [2], the conclusion of treatment by the age of 19 was made possible in five cases.

PDTA-YA data show a sharp increase in prevalence (30%) and incidence (26%) of YA with SMD, with a mean age in incident patients of 23 years, equal to the median age of the first psychotic episodes. These data suggest an improvement in the interception of YA in an initial period of their psychopathological manifestations. The data for the higher presence of young male adults confirm this result; in fact, epidemiologically, in CSMs, the female population is more represented for common mental disorders and for a higher age, while the onset of most psychiatric disorders is earlier in men, especially with regard to SMD. The percentage of incident YA patients out of total incident patients seeking help from CSM increased by 9.3%; the reduction in the mean age of total CSM incident patients (34.5 years) and the rate of 28.8% for the incident patients under the age of 30 in 2020 represent, for the PANSM guidelines, indicators that enable the early recognition of SMD to be assessed. The rate on non-Italian nationality for YA, both at the CSM (18.7%) and at Argolab2 (36.4%), is much higher than the national mean (4.6%) of foreign patients treated at the CSMs, represents another paramount indicator to assess the accessibility of the non-Italian population to mental health services [13]. Therapeutic projects and treatment profiles of an integrated type for YA with SMD increased, both in the prevalent patient population and incident patient population, by an average of 20% compared to 2018 (Figure 1). In addition, there was an overall increase in the number of interventions in both populations with SMD, with an average of 15 interventions per YA patient (Figure 2). Integrated projects and profiles consist of interventions delivered to a patient by several professionals and are an important indicator of the intensity of care provided to new patients with SMD. In 2020, the national average of interventions for this patient group was 3.1 [13] (p. 100). The administrative conclusions of treatment due to the absence of interventions—for 90 days in the DSM, for 180 in the national information system—provide valuable information on the capacity of the services to maintain a continuity in care pathways: in the YA population treatments, the conclusions of treatment due to absence of interventions for 90 days decrease by 30% (21% in 2020; national rate of 80.4% with no interventions for 180 days). It should be noted that, at the national level, the time interval without interventions that determines the conclusion of the treatment is double compared to the DSM criterion. Moreover, the rate of such conclusions equal to 0 in YA with SMD is even more significant data (Figure 3). The overall reduction in the conclusion of the treatment (24%), excluding those for an agreed termination (regular conclusion), has a direct correlation with an increase in the continuity of care pathways. In 2019 and 2020, TSO = 0; STPIT hospitalizations = 0; trend of reduction in voluntary hospitalizations in SPDC was up to 0.8% of the total, and the reduction in the mean length of stay (10 days) represent PANSM indicators that evaluate the timeliness, describe the capacity of community-based services to guarantee effective continuity of care after the hospitalization and are measures of the therapeutic efficacy of rehabilitation programs developed by CSMs [13].

In 2019/2020, repeated hospitalizations = 0 are an indicator that underline the adequacy with which the community services treat patients discharged from acute care facilities. In the last two years of the given period, diagnostic accuracy and specific treatment resulted in hospitalizations = 0 in the common mental disorders diagnostic group (Figure 5). In 2020, young adults attending the Argolab2 Potential Space averaged 25 hospitalizations and 3 repeated hospitalizations. Between 2020 and 2022, one voluntary hospitalization was needed. It should be noted that young adults attending Argolab2 suffer from the most severe symptoms and have the lowest level of social functioning compared with the total population treated at the CSM (mean Global Assessment of Functioning scale GAF score at baseline was 55). The Argolab2 intensive treatment model showed positive results: 45.4% resumed their studies; 40.9% had their first work experience; 22.7% obtained educational or training qualifications; and 18.2% lived independently (Figure 6). The resumption and conclusion of study and work paths and the achievement of housing autonomy occurred within 2 years, considering the critical period of the psychotic onset within which “Psychological and psychosocial treatments should be core elements and should be used to assist resolution of enduring positive and negative symptoms, the management of secondary comorbidity, and the promotion of recovery and positive mental health. Recovery work should emphasize the need to find meaning and develop mastery in relation to the psychotic experience” [11] (p. 123).

## 5. Conclusions

In this study, the focus on hospitalizations is not related to mere health economics or to simple adherence to the indicators. Hospitalizations, especially in this period of life, represent traumatic disruptions of the natural developmental pathways, and are mainly aimed at managing crises through pharmacological interventions. This approach misses the chance of encouraging the process of signification of psychic manifestations that, albeit in a catastrophic way, signal the need for an internal transformation, as the etymology of the word crisis teaches us. Therefore, it is of primary importance to invest in the training and capacity of the community care environment to contain crises by restoring meaning and trust in the continuity of existence. As research shows: “Antipsychotic drugs have been shown to be effective against psychotic symptoms, however, long-term therapy with antipsychotics is associated with a range of side effects, poor adherence and high rates of medication discontinuation. Most patients, even those with a good response to medication, continue to suffer from disabling residual symptoms, impaired social and occupational functioning, and a high risk of relapse” [50] (p. 2).

The presence of hospitalizations in the other disorders diagnostic group leads to reflect on the quantity of conditions that, although in absence of a severe psychiatric diagnosis, come to the attention of psychiatric wards. It is worth noting that this diagnostic group includes the pathologies that, according to the Ministry of Health, do not fall within primary psychiatric competence (substance addiction, antisocial personality disorder, moderate and severe mental retardation). Psychiatric interventions in these categories have medicolegal implications that make them aimed at the mere management of behaviors, with the result of undifferentiated and non-specific responses that represent a historical involution of psychiatry. Furthermore, diagnostic inappropriateness is likely to lead to an improper use of scarce professional resources, eliminating the possibility of investing them in effective treatment pathways. The age-specific needs of people who face their first psychotic experiences should lead to ponder on the so-called “rehabilitative” practices and on the training of those who provide them.

The word rehabilitation evokes the retrieval/restitution of skills/abilities that the individual had but which have been reduced or lost due to the mental disorder onset. However, if we reflect on the need for and importance of confrontation with the peer group as a subjectivating agent [51], on the role of school or the first work experiences, and on participation in social life, our attention necessarily shifts to something new that is about to happen in the life of the young adult, deconstructing the old model of skills achieved and then lost due to mental disorders. The is something new that the young adult could not actively access before, because still a “child”, and that then does not occur or is interrupted in the bud, due to the developmental breakdown. Therefore, the term rehabilitation is misleading.

The youth population, especially in socially disadvantaged contexts, has no access to artistic and cultural activities, necessary to guarantee a growth environment that promotes psychic development and relational skills. “Despite the growing evidence on the impact of culture on wellbeing [46,52,53] there is a recurrent argument that is raised in debates involving cultural practitioners and professionals: insisting on the role of culture in promoting wellbeing may be counterproductive in that it characterizes culture as an instrumental activity to promote other goals, however in the public interest. This argument, however, stems from a specific, and totally legitimate, vision of culture as a human activity that finds its justification in itself and has to be appreciated merely for what it is. Therefore, whereas one should avoid an over-simplification of cultural creation and participation processes as instrumental to wellbeing goals, it is equally fair to acknowledge that there may be cultural and artistic practice that may pursue such goals as part of a legitimate, historically founded vision of the role of arts and culture in contemporary societies” [54] (p. 21). Argolab2 Potential Space provides cultural and relational experiences that YA can meet and choose according to their interest and curiosity. This environment has increased confidence in the care pathway and fostered social skills, as evidenced by the data.

The academic literature underlined the terrible impact of the COVID-19 pandemic on this population and showed a correlation between the increase in suicide attempts and psychiatric disorders on the one hand and disruption to education, online teaching, and social isolation on the other hand [55,56,57,58]. This study demonstrated the efficacy of specific interventions targeting the young population and recommends that such interventions are implemented and supported by appropriate resources and facilities within DSMs. Further studies could, through statistical analysis and other qualitative data, assess the stability of the process also in relation to organizational variables.

## Figures and Tables

**Table 1:** TSMREE/CSM (17-19)—Distribution by diagnostic group. The bold indicates total.

	Referrals from TSMREE			CSM			TSMREE/CSM			Referrals to Other Services		
**Diagnostic Group**	**2018**	**2019**	**2020**	**2018**	**2019**	**2020**	**2018**	**2019**	**2020**	**2018**	**2019**	**2020**
Schizophrenia spectrum disorders	3	1	2	3	1	2	0	0	0	0	0	0
Affective psychosis	2	2	2	2	2	2	0	0	0	0	0	0
Severe personality disorders	3	3	4	3	3	4	0	0	0	0	0	0
Common mental disorders	10	2	2	10	0	0	0	1	1	0	1	1
Other disorders	1	2	2	1	0	0	0	0	0	0	2	2
No psychiatric disorder	1	3	0	1	0	0	0	3	0	0	0	0
Unknown diagnosis	1	0	0	1	0	0	0	0	0	0	0	0
Total	**21**	**13**	**12**	**21**	**6**	**8**	**0**	**4**	**1**	**0**	**3**	**3**
%	100	100	100	100	**46.2**	**66.7**	0	**30.7**	**8.3**	0	**23.1**	**25**
Mean age	**19**	**18.7**	**18.4**									

## Data Availability

The data presented in this study are available within the article and its Supplementary Materials.

## Supplementary Materials

The following supporting information can be downloaded at: https://www.mdpi.com/article/10.3390/ijerph192215252/s1, Table S1. Diagnostic groups; Table S2. Prevalent YA patients—Distribution by Gender and Diagnosis; Table S3. Prevalent YA patients—Distribution by Nationality; Table S4. Incident YA patients—Distribution by Diagnostic Group; Table S5. Incident patients—Comparison between total population and YA population; Table S6. *“Argolab2 Potential Space”*—Population.

## References

[1] McGorry P., Nordentoft M., Simonsen E. (2005). Introduction to “Early psychosis: A bridge to the future”. Br. J. Psychiatry.

[2] Ministero della Salute Definizione Dei Percorsi Di Cura Da Attivare Nei Dipartimenti Di Salute Mentale Per I Disturbi Schizofrenici, I Disturbi Dell’umore E I Disturbi Gravi Di Personalità; Accordo Conferenza Unificata. https://www.salute.gov.it/imgs/C_17_pubblicazioni_2461_allegato.pdf.

[3] Kessler R.C., Amminger G.P., Aguilar-Gaxiola S., Alonso J., Lee S., Ustün T.B. (2007). Age of onset of mental disorders: A review of recent literature. Curr. Opin. Psychiatry.

[4] Kirkbride J.B., Hameed Y., Ankireddypalli G., Ioannidis K., Crane C.M., Nasir M., Kabacs N., Metastasio A., Jenkins O., Espandian A. (2017). The Epidemiology of First-Episode Psychosis in Early Intervention in Psychosis Services: Findings From the Social Epidemiology of Psychoses in East Anglia [SEPEA] Study. Am. J. Psychiatry.

[5] Penttilä M., Jääskeläinen E., Hirvonen N., Isohanni M., Miettunen J. (2014). Duration of untreated psychosis as predictor of long-term outcome in schizophrenia: Systematic review and meta-analysis. Br. J. Psychiatry.

[6] Schimmelmann B.G., Huber C.G., Lambert M., Cotton S., McGorry P.D., Conus P. (2008). Impact of duration of untreated psychosis on pre-treatment, baseline, and outcome characteristics in an epidemiological first-episode psychosis cohort. J. Psychiatr. Res..

[7] Ministero della Salute Piano di Azioni Nazionale per la Salute Mentale. https://www.salute.gov.it/imgs/C_17_pubblicazioni_1905_allegato.pdf.

[8] Singh S.P., Evans N., Sireling L., Stuart H. (2005). Mind the gap: The interface between child and adult mental health services. Psychiatr. Bull..

[9] Signorini G., Singh S.P., Boricevic-Marsanic V., Dieleman G., Dodig-Ćurković K., Franic T., Gerritsen S.E., Griffin J., Maras A., McNicholas F. (2017). Architecture and functioning of child and adolescent mental health services: A 28-country survey in Europe. Lancet Psychiatry.

[10] Paul M., O’Hara L., Tah P., Street C., Maras A., Ouakil D.P., Santosh P., Signorini G., Singh S.P., Tuomainen H. (2018). A systematic review of the literature on ethical aspects of transitional care between child-and adult-orientated health services. BMC Med. Ethics.

[11] International Early Psychosis Association Writing Group (2005). International clinical practice guidelines for early psychosis. Br. J. Psychiatry Suppl..

[12] Saraceno B. (2001). Liberare le Identità: Dalla Riabilitazione Psicosociale Alla Possibile Cittadinanza.

[13] (2020). Ministero Della Salute, Analisi Dei Dati del Sistema Informativo per la Salute Mentale (SISM) Anno. https://www.salute.gov.it/imgs/C_17_pubblicazioni_3212_allegato.pdf.

[14] World Health Organization, Mental Health Action Plan. 2013–2020. https://www.who.int/publications/i/item/9789241506021.

[15] Yung Alison R., Cotter J., Mcgorry P.D. (2020). Youth Mental Health Approaches to Emerging Mental Ill-Health in Young People.

[16] ROMA https://www.comune.roma.it/web/it/roma-statistica-servizi-sociali1.page.

[17] Singh S.P., Anderson B., Liabo K., Ganeshamoorthy T., Guideline Committee (2016). Supporting young people in their transition to adults’ services: Summary of NICE guidance. BMJ.

[18] Beckmann D., Lowman K.L., Nargiso J., McKowen J., Watt L., Yule A.M. (2020). Substance-induced Psychosis in Youth. Child. Adolesc. Psychiatr. Clin. N. Am..

[19] Winnicott D.W. (2016). The Use of the Word “Use”. The Collected Works of D.W. Winnicott, Volume 8, 1967–1968.

[20] Blos P. (1967). The second individuation process of adolescence. Psychoanal. Study Child..

[21] Arain M., Haque M., Johal L., Mathur P., Nel W., Rais A., Sandhu R., Sharma S. (2013). Maturation of the adolescent brain. Neuropsychiatr. Dis. Treat..

[22] Giedd J.N. (2015). The amazing teen brain. Sci. Am..

[23] Deleuze G. (2020). La soggettivazione. Corso su Michel Foucault (1985–1986).

[24] Cahn R., Gutton P., Robert P., Tisseron S. (2013). L’ado Et son psy, Nouvelles Approches Thérapeutiques en Psychanalyse.

[25] Blos P. (1962). On Adolescence: A Psychoanalytic Interpretation.

[26] O’Connor K., Muller Neff D., Pitman S. (2018). Burnout in mental health professionals: A systematic review and meta-analysis of prevalence and determinants. Eur. Psychiatry.

[27] Forstag E.H., Cuff P.A. (2018). National Academies of Sciences, Engineering, and Medicine; Health and Medicine Division; Board on Global Health.

[28] Bodenheimer T., Sinsky C. (2014). From triple to quadruple aim: Care of the patient requires care of the provider. Ann. Fam. Med..

[29] Braithwaite J., Herkes J., Ludlow K., Testa L., Lamprell G. (2017). Association between organisational and workplace cultures, and patient outcomes: Systematic review. BMJ Open.

[30] Gómez-Revuelta M., Pelayo-Terán J.M., Juncal-Ruiz M., Vázquez-Bourgon J., Suárez-Pinilla P., Romero-Jiménez R., Setién Suero E., Ayesa-Arriola R., Crespo-Facorro B. (2020). Antipsychotic Treatment Effectiveness in First Episode of Psychosis: PAFIP 3-Year Follow-Up Randomized Clinical Trials Comparing Haloperidol, Olanzapine, Risperidone, Aripiprazole, Quetiapine, and Ziprasidone. Int. J. Neuropsychopharmacol..

[31] Sherrill J., Amy B., Susan G., Azrin T. Evidence-Based Treatments for First Episode Psychosis: Components of Coordinated Specialty Care. NIMH. https://www.nimh.nih.gov/sites/default/files/documents/health/topics/schizophrenia/raise/nimh-white-paper-csc-for-fep.pdf.

[32] Robinson D.G., Schooler N.R., Correll C.U., John M., Kurian B.T., Marcy P., Miller A.L., Pipes R., Trivedi M.H., Kane J.M. (2018). Psychopharmacological Treatment in the RAISE-ETP Study: Outcomes of a Manual and Computer Decision Support System Based Intervention. Am. J. Psychiatry.

[33] Haram A., Fosse R., Jonsbu E., Hole T. (2019). Impact of Psychotherapy in Psychosis: A Retrospective Case Control Study. Front. Psychiatry.

[34] Fusar-Poli P., Papanastasiou E., Stahl D., Rocchetti M., Carpenter W., Shergill S., McGuire P. (2015). Treatments of Negative Symptoms in Schizophrenia: Meta-Analysis of 168 Randomized Placebo-Controlled Trials. Schizophr. Bull..

[35] Skelton M., Khokhar W.A., Thacker S.P. (2015). Treatments for delusional disorder. Cochrane Database Syst. Rev..

[36] Gergov V., Milic B., Löffler-Stastka H., Ulberg R., Vousoura E., Poulsen S. (2022). Psychological Interventions for Young People With Psychotic Disorders: A Systematic Review. Front. Psychiatry.

[37] Goodyer I.M., Reynolds S., Barrett B., Byford S., Dubicka B., Hill J., Holland F., Kelvin R., Midgley N., Roberts C. (2017). Cognitive behavioural therapy and short-term psychoanalytical psychotherapy versus a brief psychosocial intervention in adolescents with unipolar major depressive disorder (IMPACT): A multicentre, pragmatic, observer-blind, randomised controlled superiority trial. Lancet Psychiatry.

[38] Nemirovski Edlund J., Carlberg G. (2016). Psychodynamic psychotherapy with adolescents and young adults: Outcome in routine practice. Clin. Child. Psychol. Psychiatry.

[39] Gerber A.J., Fonagy P., Bateman A., Higgitt A. (2004). Structural and symptomatic change in psychoanalysis and psychodynamic psychotherapy of young adults: A quantitative study of process and outcome. J. Am. Psychoanal. Assoc..

[40] Škodlar B., Henriksen M.G., Sass L.A., Nelson B., Parnas J. (2013). Cognitive-Behavioral Therapy for Schizophrenia: A Critical Evaluation of Its Theoretical Framework from a Clinical-Phenomenological Perspective. Psychopathology.

[41] Lysaker P.H., Lysaker J.T. (2010). Schizophrenia and alterations in self-experience: A comparison of 6 perspectives. Schizophr. Bull..

[42] Bendall S., Allott K., Jovev M., Marois M.J., Killackey E.J., Gleeson J.F., Alvarez-Jimenez M., McGorry P.D., Jackson H.J. (2015). Therapy contamination as a measure of therapist treatment adherence in a trial of cognitive behaviour therapy versus befriending for psychosis. Behav. Cogn. Psychother..

[43] Lelo K., Monni S., Tomassi F. (2019). Le Mappe Della Disuguaglianza. Una Geografia Sociale Metropolitana.

[44] Iyer S., Mustafa S., Gariépy G., Shah J., Joober R., Lepage M., Malla A. (2018). A NEET distinction: Youths not in employment, education or training follow different pathways to illness and care in psychosis. Soc. Psychiatry Psychiatr. Epidemiol..

[45] Rosenheck R., Mueser K.T., Sint K., Lin H., Lynde D.W., Glynn S.M., Robinson D.G., Schooler N.R., Marcy P., Mohamed S. (2017). Supported employment and education in comprehensive, integrated care for first episode psychosis: Effects on work, school, and disability income. Schizophr. Res..

[46] Fancourt D., Finn S. (2019). What Is the Evidence on the Role of the Arts in Improving Health and Well-Being? A Scoping Review.

[47] Winnicott D.W. (1971). Playing and Reality.

[48] Graves R. (1993). The Golden Fleece.

[49] Lega I., Pelletier J.-F., Caroppo E. (2022). Editorial: Long term psychiatric care and COVID-19. Front. Psychiatry.

[50] Guo X., Zhai J., Liu Z., Fang M., Wang B., Wang C., Hu B., Sun X., Lv L., Lu Z. (2010). Effect of antipsychotic medication alone vs combined with psychosocial intervention on outcomes of early-stage schizophrenia: A randomized, 1-year study. Arch. Gen. Psychiatry.

[51] Guénoun T., Attigui P. (2021). The therapeutic group in adolescence: A process of intersubjectivation. Int. J. Psychoanal..

[52] Zarobe L., Bungay H. (2017). The role of arts activities in developing resilience and mental wellbeing in children and young people a rapid review of the literature. Perspect. Public Health.

[53] Arts and Health: Supporting the Well-Being of Forcibly Displaced People. https://bci-hub.org/documents/arts-and-health-supporting-well-being-forcibly-displaced-people.

[54] Workshop for the Experts of the EU Member States on Culture for Social Cohesion Outcomes and Lessons Learned, EC Directorate-General for Education, Youth, Sport and Culture-Unit for Cultural Policy. Report-Workshop for EU Experts on Culture for Social Cohesion|Culture and Creativity. https://culture.ec.europa.eu/document/report-workshop-for-eu-experts-on-culture-for-social-cohesion.

[55] Suicide Increasing Amongst Europe’s Youth, Governments Underprepared. https://www.euractiv.com/section/coronavirus/news/suicide-increasing-amongst-europes-youth-governments-underprepared/.

[56] Winter R., Lavis A. (2021). The Impact of COVID-19 on Young People’s Mental Health in the UK: Key Insights from Social Media Using Online Ethnography. Int. J. Environ. Res. Public Health.

[57] Lee J. (2020). Mental health effects of school closures during COVID-19. Lancet Child. Adolesc. Health.

[58] McKinlay A.R., May T., Dawes J., Fancourt D., Burton A. (2021). “You’re just there, alone in your room with your thoughts” A qualitative study about the impact of lockdown among young people during the COVID-19 pandemic. medRxiv.

