# Supplementary material for: A Treatment Model for Young Adults with Severe Mental Disorders in a Community Mental Health Center: The Crisalide Project and the Potential Space
Source: Int J Environ Res Public Health. 2022 Nov 18;19(22):15252. doi: 10.3390/ijerph192215252 (PMC9690010; doi:10.3390/ijerph192215252)
Supplement: Supplementary file 1 [file ijerph-19-15252-s001.zip › ijerph-1910867-supplementary.pdf]

Table S1. Diagnostic groups

DIAGNOSTIC GROUP	ICD-9-CM
Schizophrenia Spectrum Disorders	In range from 295-295.99, or 297-299.99
Affective Psychosis	In range from 296-296.99
Severe Personality Disorders	In range from 301-301.2, or 301.83
Common Mental Disorders	In range from 301.4-301.6, or 301.8-301.82, or 306-309.99, or 311, or 316.99
Other disorders	In range from 290-294.99, or 303-305.99, or 301.7, or 301.84-301.99, or 310-310.99, or 312-315.99, or 317-319.99
No psychiatric disorder	V70.1 or V70.2 or V70.9 or V71.09 or V79.0
Unknown diagnosis	

Table S2. Prevalent YA patients – Distribution by Gender and Diagnosis

DIAGNOSTIC GROUP	2018						2019						2020					
	M		F		TOT		M		F		TOT		M		F		TOT	
	N	%	N	%	N	%	N	%	N	%	N	%	N	%	N	%	N	%
Schizophrenia Spectrum Disorders	58	74,4	20	25,6	78	27,4	41	67,2	20	32,8	61	39,1	34	65,4	18	34,6	52	42,9
Affective Psychosis	21	53,8	18	46,2	39	13,7	15	46,8	17	53,1	32	20,5	10	43,5	13	56,5	23	19,1
Severe Personality Disorders	13	54,2	11	45,8	24	8,4	10	47,6	11	52,4	21	13,5	12	57,1	9	42,8	21	17,3
Common Mental Disorders	61	50,4	60	49,6	121	42,4	18	54,5	15	45,5	33	21,1	12	60	8	40	20	16,5
Other disorders	15	78,9	4	21,1	19	6,6	5	71,4	2	28,6	7	4,5	2	40	3	60	5	4,1
No psychiatric disorder	0	0	2	100	2	0,7	1	50	1	50	2	1,3	0	0	0	0	0	0
Unknown diagnosis	0	0	2	100	2	0,7	0	0	0	0	0		0	0	0	0	0	
TOTAL by gender	168		117		285	100	90		66		156		70		51		121	
TOTAL PATIENTS WITH SMD by gender	92	32,3	49	17,2	141	49,5	66	57,9	48	42,1	114	73,1	56	58	40	42	96	79,3

Table S3. Prevalent YA patients – Distribution by Nationality

NATIONALITY	2018		2019		2020	
	N	%	N	%	N	%
Italians	231	81,1	125	80,1	100	82,6
Non-Italians	54	18,9	31	19,9	21	17,4
TOTAL	285	100	156	100	121	100

Table S4. Incident YA patients – Distribution by Diagnostic Group

DIAGNOSTIC GROUP	2018		2019		2020	
	**N**	**%**	**N**	**%**	**N**	**%**
Schizophrenia Spectrum Disorders	18	18,9	12	34,3	12	31,6
Affective Psychosis	10	10,5	7	20	6	15,8
Severe Personality Disorders	8	8,4	4	11,4	5	13,2
Common Mental Disorders	56	58,9	10	28,6	12	31,5
Other disorders	1	1,1	0	0	3	7,9
No psychiatric disorder	1	1,1	2	5,7	0	0
Unknown diagnosis	1	1,1	0	0	0	0
TOTAL	**95**	100	**35**	100	**38**	100
TOTAL PATIENTS WITH SMD	36	**37,8**	23	**65,7**	23	**60,6**

Table S5. Incident patients – Comparison between total population and YA population

INCIDENT PATIENTS	2018	2019	2020
TOTAL POPULATION	488	142	132
YA POPULATION	95	35	38
%	**19,5**	**24,7**	**28,8**
**INCIDENT PATIENTS WITH SMD**	**2018**	**2019**	**2020**
TOTAL POPULATION	183	99	89
YA POPULATION	36	23	23
%	**19,7**	**23,2**	**25,8**
**MEAN AGE INCIDENT PATIENTS**	**2018**	**2019**	**2020**
TOTAL POPULATION	45	43	**34,5**
YA POPULATION	23	23	**23**

Table S6. *"Argolab2 Potential Space"* – Population

DIAGNOSTIC GROUP	MALE	FEMALE	TOTAL	%
Schizophrenia Spectrum Disorders	6	5	11	50,0
Affective Psychosis	2	3	5	22,7
Severe Personality Disorders	1	5	6	27,3
TOTAL	9	13	22	
%	41%	59%		100%
NATIONALITY			N.	%
ITALIANS			14	63,6
